# A Novel Dependoparvovirus Identified in Cloacal Swabs of Monk Parakeet (*Myiopsitta monachus*) from Urban Areas of Spain

**DOI:** 10.3390/v15040850

**Published:** 2023-03-26

**Authors:** Christian Sánchez, Ana Doménech, Esperanza Gomez-Lucia, José Luis Méndez, Juan Carlos Ortiz, Laura Benítez

**Affiliations:** 1 Department of Genetics, Physiology and Microbiology, Faculty of Biological Sciences, Complutense University of Madrid (UCM), C. de José Antonio Novais, 12, 28040 Madrid, Spain; 2 Department of Animal Health, Veterinary Faculty, Complutense University of Madrid, Av. Puerta de Hierro, s/n, 28040 Madrid, Spain; 3 Research Group, “Animal viruses” of Complutense University of Madrid, 28040 Madrid, Spain; 4 UTE Control. C/ Federico Salmón 13, 28016 Madrid, Spain; 5 Department of Fauna and Biodiversity, Ayuntamiento de Madrid, 28045 Madrid, Spain

**Keywords:** metagenomics, phylogenetics, dependoparvovirus, invasive species, psittacid

## Abstract

The introduction of invasive birds into new ecosystems frequently has negative consequences for the resident populations. Accordingly, the increasing population of monk parakeets (*Myiopsitta monachus*) in Europe may pose a threat because we have little knowledge of the viruses they can transmit to native naïve species. In this study, we describe a new dependoparvovirus detected by metagenomic analysis of cloacal samples from 28 apparently healthy individuals captured in urban areas of Madrid, Spain. The genomic characterization revealed that the genome encoded the NS and VP proteins typical of parvoviruses and was flanked by inverted terminal repeats. No recombination signal was detected. The phylogenetic analysis showed that it was closely related to a parvovirus isolated in a wild psittacid in China. Both viruses share 80% Rep protein sequence identity and only 64% with other dependoparvoviruses identified in Passeriformes, Anseriformes, and Piciformes and are included in a highly supported clade, which could be considered a new species. The prevalence was very low, and none of the additional 73 individuals tested positive by PCR. These results highlight the importance of exploring the viral genome in invasive species to prevent the emergence of novel viral pathogenic species.

## 1. Introduction

The introduction of invasive species of birds into new ecosystems has several negative consequences including such as declines in biodiversity [1]. In addition, it is well-known that the introduction of new pathogens such as viruses can infect native naïve species and cause high economic losses due to the spread of new diseases and the rapid increase of zoonotic risk that poses a significant threat to global public health [2]. Numerous novel viruses or variants of different species have been described in birds in recent years [3]. The use of metagenomics for the study of viral communities, which benefits from the sensitivity of next-generation sequencing (NGS), is a useful tool for the identification of viruses in diverse environments and is particularly interesting for detecting viral sequences in apparently healthy birds [4]. Monk parakeets (*Myiopsitta monachus*) are one of the most studied examples of invasive exotic avian species in urban areas [5,6], capable of displacing native species such as sparrows and blackbirds and causing concern to official veterinary services and human public health, as has been described in the city of Madrid, Spain. Monk parakeets were first observed in Madrid in 1985, and since then, the monitoring and managing programs of invasive species have reported an increase of 31% between 2005 and 2015, with the population doubling every 4.5 years [7]. The uncontrolled increase of their population and the potential risk for avian and human health requires a better understanding of the possibility of pathogen transmission, especially by apparently healthy monk parakeets. The study of their fecal viral geome is essential to achieving this objective since it is one of the main routes of pathogen transmission to the environment or to other birds that share their habitat.

Several viral diseases have been detected in both wild and captive psittaccid populations worldwide. Three DNA viruses that cause severe morbidity and mortality in captive collections of psittacine birds are beak and feather disease virus (BFDV), a member of the family *Circoviridae*, *Psittacid alphaherpesvirus 1* (PsHV1) which causes Pacheco’s disease, and *Aves polyomavirus 1* (APV), the agent of budgerigar fledgling disease [8]. However, field surveys in free-ranging psittacines (*Pssitacula* and *Myiopsitta*) from some areas of Western Europe show a low prevalence of bornaviruses and polyomaviruses and contradictory results for BFDV, although no transmission of circoviruses to native birds has been detected [9,10,11].

Another DNA family that may infect birds is *Parvoviridae*, which are generally associated with gastrointestinal disease. They are widely spread in countries with a large poultry industry, causing high mortality, watery diarrhea, lethargy, anorexia, and prostration in chickens, turkeys, geese, and ducks [12]. Avian parvoviruses are included in the subfamily *Parvovirinae*, genus *Aveparvovirus* (three species) and genus *Dependoparvovirus* which contains 10 species, of which only two infect birds [13], *Anseriform dependoparvovirus 1* formerly known as Duck dependoparvovirus 1 (DPV), and *Avian dependoparvovirus 1* (AAAV). DPV includes well-known pathogenic viruses, such as Goose parvovirus (GPV) and Muscovy duck parvovirus (MDPV), while AAAV is non-pathogenic [14]. *Hamaparvovirinae*, a new subfamily recently recognized, includes a new genus, *Chaphamaparvovirus*, with 16 species, six of which have been identified in avian hosts [13,15]. Several additional avian viruses showing homology to the subfamily *Densovirinae* have been described [15].

The particles of dependoparvoviruses are non-enveloped, icosahedral, and 23–28 nm in diameter. The genome consists of single-stranded DNA of 4–6 kb and the genomic organization of the virus is composed of two major open reading frames (ORF) called Rep (replication protein) and VP (viral protein) [15,16]. The replication protein is involved in the initiation of replication and packaging of viral DNA, while VP forms the capsid and is associated with the processes of viral entry, capsid assembly, and DNA packaging. Usually, genomes are flanked by palindromic inverted terminal repeat (ITR) hairpins, which contain signals and binding sites for the initiation of replication, and adeno-associated virus type 2 has a role in the integration into the host genome [17].

The free circulation of potentially pathogenic viruses in invasive populations of *M. monachus* is currently not well-studied, representing a potential threat of infection to native species or even of zoonotic risk to the human population. In this study, we have identified by NGS a novel dependoparvovirus in cloacal swabs of monk parakeets in urban areas of the city of Madrid, Spain. We describe its genomic organization, phylogenetical analysis, and prevalence, considering the species described in the family and the similarities with other dependoparvoviruses identified in psittacines.

## 2. Materials and Methods

### 2.1. Sample Collection

Sampling was carried out in November 2021 in Madrid during the official population control campaign done by the local Department of Fauna and Biodiversity of Madrid City Council. Twenty-eight cloacal samples were taken from adult specimens of *M. monachus* using ethylene oxide-sterile swabs (Nerbe plus, Winse, Germany), which were placed in 1.5 mL Eppendorf tubes with 300 µL of BD universal viral transport medium (VTM) (Becton Dickinson, Sparks, MD, USA), transported at 4 °C and stored at −20 °C.

### 2.2. Sample Treatment, DNA Isolation, and Metagenomic Analysis

The 28 cloacal swab samples were vortexed and squeezed to release epithelial cells. They were combined in groups of four to make seven pools, each containing 200 µL of the VTM of each sample for a total of 800 µL/pool. The processing of each group consisted in a centrifugation at 8000 rpm for 5 min and both the sediment and the supernatant were processed. The supernatant was filtered through 0.45 µm pore-sized column filters (Ultrafree-MC-HV; Millipore, Cork, Ireland) to reduce the bacterial load and other contaminants. The sediment from each pool was resuspended in 50 µL of PBS buffer, subjected to three freeze-thaw cycles at −80 °C, mixed, and centrifuged at 13,000 rpm for 1 min. These supernatants were also filtered, pooled with the supernatants from the first centrifugation, and treated with a mixture of nucleases, Turbo DNase, (Invitrogen, Waltham, United States), Benzonase (Novagen), and RNase I (Thermo Fisher Scientific, Vilnius, Lithuania) to digest unprotected nucleic acids. The DNA was extracted using the QIAmp DNA Mini kit (Qiagen, Hilden, Germany) and eluted in 75 µL per group, which were pooled together (525 µL). This volume was concentrated to 64 µL in a SpeedVac (Eppendorf Iberica, Madrid, Spain) for 100 min. The concentration was measured by spectrophotometry (ND-1000 Thermo Fisher Scientific) and fluorometry (Qubit).

The metagenomic analysis was performed by the FISABIO Research Center in Valencia (Spain) using the concentrated pool corresponding to all 28 cloacal swabs. The libraries were generated using the QIAseq FX DNA Library Kit (Qiagen) and sequenced using 600-cycle MiSeq reagent kits (Illumina, San Diego, CA, USA) on a MiSeq platform. The generated raw reads were qualitatively checked, filtered using FastQC v0.119 [18], and trimmed with Trimmomatic v0.39 [19]. The high-quality reads were de novo assembled using SqueezeMeta pipeline v1.6.0 [20] and compared with a non-redundant and viral genome and proteome database using BLASTx with a cut-off E-value of 0.001. The sequence was stored in GenBank with the accession number OQ101836.

### 2.3. Genomic Characterization and Recombination Analysis

The sequence was analyzed with the Lasergene computerized sequence analysis software (Dnastar Inc., Madison, WI, USA). The determination of the ORFs was conducted with ORFfinder [21] and the prediction of DNA folding with the UNAFold Web Server [22]. Inverted terminal repeats were detected using the EMBOSS explorer Einverted available at http://emboss.toulouse.inra.fr/cgi-bin/emboss/einverted (accessed on 22 March 2023). Promoter sequences were mapped with Neural Network Promoter Prediction [23]. RNA splicing donors and acceptors were detected using NetGene2 v2.42 [24]. Polyadenylation sites were identified with PolyApred [25] and NLS (nucleolar localization signal) prediction with NoD [26]. The detection of recombination signals was performed by RDP, GENCONV, Chimaera, MaxChi, BootScan, SiScan, and 3Seq methods using the RDP5 v5.35 software [27].

### 2.4. Phylogenetic Analysis and Species Demarcation

The phylogenetic analysis of the dependoparvovirus was based on the amino-acid sequences of the Rep protein. The sequences were aligned using MAFFT online software services [28]. Best-fit substitution models for phylogenetic analysis were estimated with SMS: Smart Model Selection in PhyML [29], with Akaike Information Criteria (AIC). To infer phylogenetic relationships among the sequences, Maximum Likelihood trees were reconstructed with PhyML version 3 [30] using a Q.pfam+R+F amino-acid substitution model, and nodal supports were estimated with 1000 bootstrap replicates. Trees were visualized using iTOL version 6 [31]. The species demarcation was performed using a sequence pairwise identity matrix using SDT version 1.2 [32].

### 2.5. DNA Extraction and Screening of Dependoparvovirus in M. monachus

To study the prevalence of Sp_PsDPV in the *M. monachus* population in Madrid, cloacal swab samples were taken from another 73 individuals captured between November 2021 and March–June 2022. The DNA was extracted from 200 µL of the sample using the QIAamp DNA Mini Kit (Qiagen) according to the instructions of the manufacturer. The molecular detection of dependoparvovirus was performed by PCR using the primers DpvF-01 (5′-TCCGGTCATCATTACGAGCAACAC-3′) and DpvR-01 (5′-GCGGAGCAATAGAAACGAACCTTA-3′). The PCR volume was 25 µL and contained 2.5 µL of PCR Buffer 10× (Applied Biosystems, Warrington, UK), 2.5 mM of MgCl_2_ (Invitrogen), 0.8 mM of each deoxynucleotide diphosphate (Metabion, Planegg, Germany), 50 pmol/µL of each primer, 0.625 U of AmpliTaq DNA Polymerase (Applied Biosystems, Foster City, CA, USA), and 5 µL of DNA sample (diluted 10^−1^). The PCR conditions were denaturation at 95 °C for 4 min, followed by 35 cycles of denaturation at 95 °C for 40 s, annealing at 53 °C for 30 s, extension at 72 °C for 40 s, and final extension at 72 °C for 7 min.

## 3. Results

### 3.1. Sequence, Genomic Organization, and Recombination Analysis

**Figure 1:**
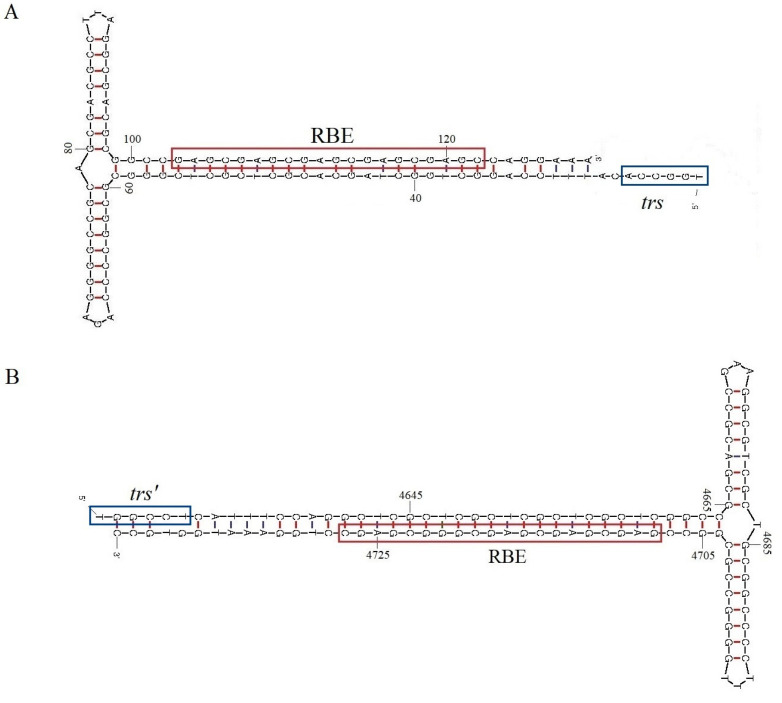
Inverted terminal repeats of the 5′ (**A**) and 3′ (**B**) termini of Sp_PsDPV. The Rep Binding Element (RBE) and the Terminal Resolution Site (trs) are enclosed in boxes.

The NGS from the pool of 28 cloacal swabs of Myiopsitta monachus resulted in 21,925,378 total reads, of which 18,730 (0.09%) were viral reads. Of these viral reads, 1028 (5.5%) corresponded to the Parvoviridae and included a sequence identified as a dependoparvovirus. The complete genome length of this parvovirus, provisionally named as Spain psittacid dependoparvovirus (Sp_PsDPV), was 4865 nucleotides (nt) and encoded two ORFs, one for Repand and another for the VP, with an intergenic region of two nucleotides. The sequence was flanked by ITRs of 244 nt and contained 16-bp double-stranded DNA, which formed typical T-shaped hairpins/structures (Figure 1). A Rep binding element (RBE) was present in both terminal repeat (TR) sequences consisting of five imperfect repetitions of GAGY. In addition, a trs (terminal resolution site) as part of the packing signal, identified as TGGCCA [33], was located upstream of the hairpins (Figure 1).

**Figure 2:**
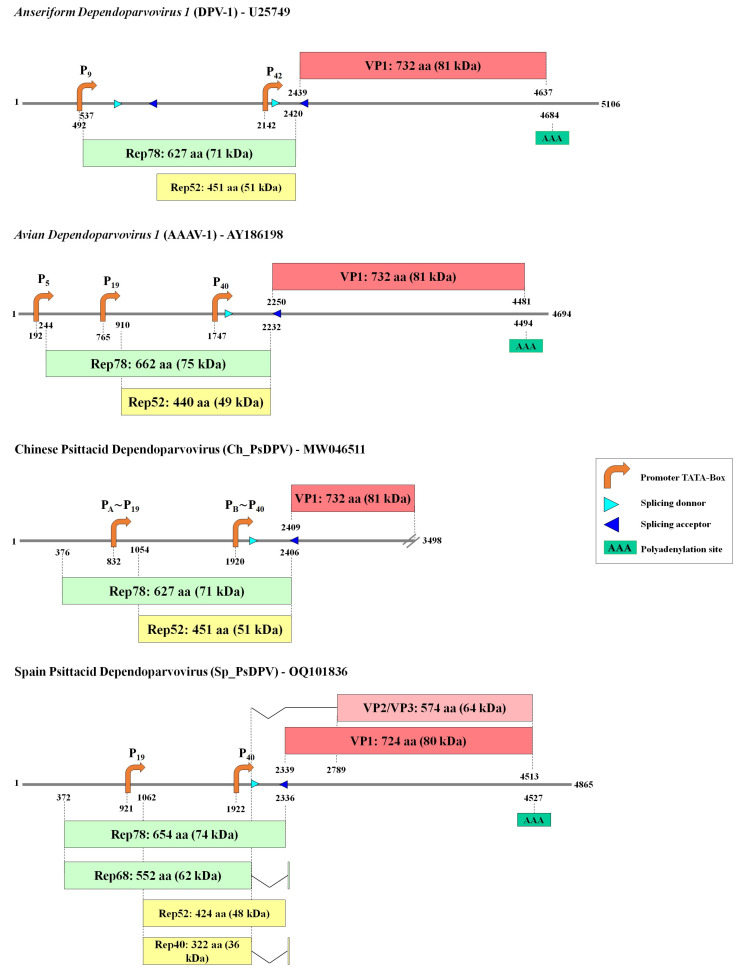
Genomic organization of Anseriform Dependoparvovirus 1 (25749), Avian Dependoparvovirus 1 (AY186198), and the dependoparvoviruses isolated from Psittacidae (Sp_PsDPV and Ch_PsDPV). The numbers shown under the bars that represent the genomes correspond to the nt position. The promoters of Ch_PsDPV were named P_A_∼P_19_ (Promoter A, such as P_19_) and P_B_∼P_40_ (Promoter B, such as P_40_).

The genomic organization was analyzed in comparison to the closest sequence in GenBank, a partial psittacidae dependoparvovirus sequence from China identified in a metagenomic analysis (Accession number MW046511) [34], hereafter named Ch_PsDPV (Figure 2). Rep genes are 1965 (Sp_PsDPV) and 2031 nt (Ch_PsDPV) long, encoding replication initiator proteins (Rep) of 654 and 676 aa, respectively. Conserved Rep motifs were identified in both sequences and compared to those of the two avian species of the Dependoparvovirus genus, Anseriform dependoparvovirus 1 (DPV) (U22967) and Avian dependoparvovirus 1 (AAAV) (AY186198). The rolling-circle replication (RCR) motifs [34], RCR II (YF**H**L**H**VLL/FE/Q), and RCR III (**Y**LIP**K**), were located at the N-terminus of Rep with slight variations in both proteins (conserved amino acids are in bold). The Walker motifs [35,36] for helicase superfamily 3 (SF3) were identified around positions 344 to 430 aa in Sp_PsDPV and 338 to 425 in Ch_PsDPV as Walker A (**G**PATT**GKT**), Walker B (IIWW**EE**), Walker B’ (**K**AILGGSRVRVDQ**K**), and Walker C (PVIITSN).

The VP ORF of Sp_PsDPV was 2175 nucleotides (724 aa) but could not be compared to the Ch_PsDPV, which is a partial ORF. The N-terminal region of VP contains the Calcium-binding loop (62-**GP**F**N**-65) and the Phospholipase A2 (PLA2) catalytic (76-**D**AAALE**HD**KA**YD**-87) motifs [37]. Additionally, a monopartite NLS (nucleolar localization signal) was identified (155-VDDFFPKKKKAKTNHTEPEKSS-176), which is highly similar to several sequences within the AAAV (Supplementary Table S1).

Three potential promoters were identified in both sequences, along with a canonical polyadenylation signal in Sp_PsDPV at position 4527 (Supplementary Table S2). Promoters P_19_ and P_40_ were located at positions 921 and 1922 for Sp_PsDPV and 832 and 1920 for Ch_PsDPV and were composed of canonical TATA boxes and located in the expected regions. Nevertheless, unlike the type species DPV and AAAV, no canonical promoter was located upstream of the Rep ORF for either of the two psittacine viruses. However, a putative CCAAT-box (133-GGCCAATCT-141; Figure 1) was located downstream of the RBE sequence in Sp_PsDPV. Since in more than 90% of mammalian promoters transcription-independent initiation starts in a CpG island [38], we analyzed the 230 nt sequence upstream of the Rep ORF. Five different transcription start sites were predicted, which were 99, 115, 131, 158, and 192 nt downstream from the CCAAT-box, all of them with a low score. However, the G+C content of the upstream regions was over 52% and the CpG density calculated as over expected (OE) ratio (OE = [number of CpGs/{number of Cs × number of Gs}] × length of the region in nucleotides) [39] were above 0.6 (0.7, 0.8, 0.8, 0.96, and 1.05, respectively) which could suggest that it may function as a CpG promoter. We did not observe these features in the sequence Ch_PsDPV.

Like the other members of the genus Dependoparvovirus, a potential intron was identified in the middle of the genome with the splicing donor and the splicing acceptor at the positions 2018/2351 in Sp_PsDPV and 2049/2460 in Ch_PsDPV (Supplementary Table S2). The combined use of P_5_ and P_19_ and alternative splicing might give the characteristic ancillary proteins that have been named Rep. The promoter P_5_ could activate the transcription of unspliced and spliced mRNA encoding proteins of 654 aa (74 KDa) and 552 aa (62 KDa) which may correspond to the proteins Rep78 and Rep68, respectively. The promoter P_19_ might generate transcripts encoding a protein of 424 aa (48 KDa) from unspliced mRNA, similar to Rep52, or a protein of 322 aa (36 KDa), similar to Rep40, using alternative splicing. Both proteins lack the N-terminal endonuclease/RBS-binding domain. The third promoter, P_40_, could activate the expression of the different capsid proteins. The translation of unspliced mRNA might generate the longer protein, VP1 (724 aa), and a shorter protein counterpart, VP2/VP3 (528 aa), using the internal ATG at position 2930 by splicing.

No recombination signals were detected in the sequences of Sp_PsDPV and Ch_PsDPV using an alignment of 62 complete and partial genomes of dependoparvoviruses.

### 3.2. Phylogenetic and Species Demarcation Analysis

**Figure 3:**
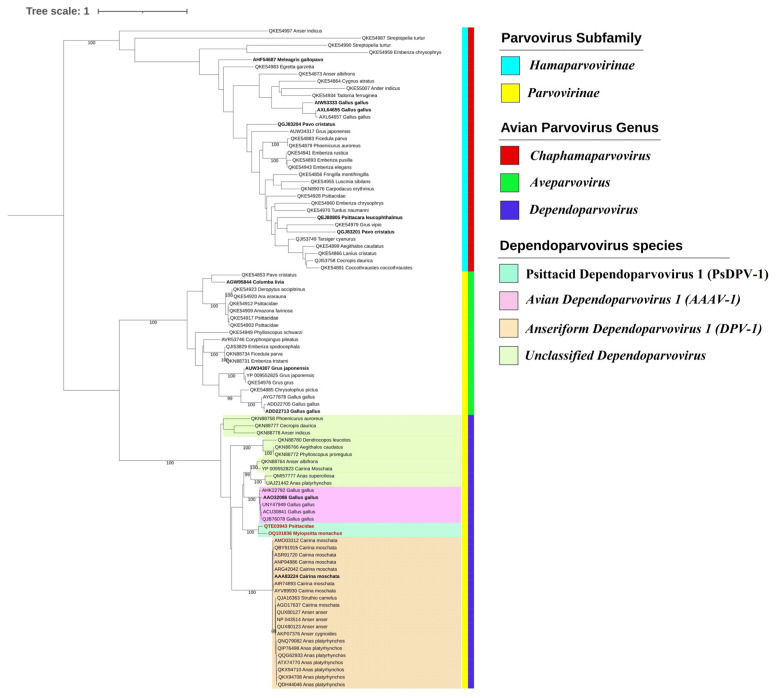
Maximum-likelihood phylogenetic inference of 92 Rep protein sequences of avian parvoviruses. The tree was inferred under a Q.pfam+R+F amino acid substitution model with nodal support values based on 1000 bootstrap replicates. The color code for the subfamilies, genera, and species used in the vertical bars to the right of the names of the species in the tree is indicated in the legend on the right. Representative sequences of avian parvovirus species are highlighted in bold, and the dependoparvoviruses from Psittacidae are highlighted in red. Only the support values above 98 are displayed.

The phylogenetic analysis based on 92 full-length Rep protein sequences revealed that avian parvoviruses are divided into two main clusters corresponding to members of the subfamilies Hamaparvovirinae and Parvovirinae (Figure 3). The genera Aveparvovirus and Dependoparvovirus are highly supported, and the species Anseriform dependoparvovirus 1 (DPV), recognized by the International Committee on Taxonomy of Viruses (ICTV) and which includes the well-known pathogenic GPV and MDPV, forms a well-supported cluster. The sequences more closely related to the species Avian dependoparvovirus 1 (AAAV) are more divergent and include several unclassified dependoparvovirus. As expected, Sp_PsDPV is most closely related to Ch_PsDPV (QTE03943) and formed another distinct clade (shown in green in Figure 3).

The analysis of the identity of Rep proteins of avian dependoparvovirus (Supplementary Figure S1) confirmed that the dependoparvovirus identified in *M. monachus* in Spain shared approximately 80% of its identity with the virus of Chinese psittacidae and less than 64% with other Rep proteins from dependoparvoviruses from chickens or passerine birds. Likewise, the identity analysis of the VP1 protein corroborates the similarity between both sequences, which share approximately 80% of their identities and less than 74% with viruses identified in birds from different orders, mainly Galliformes, Anseriformes, Piciformes, and Passeriformes.

### 3.3. Screening of Dependoparvovirus in Monk Parakeet

The presence of Sp_PsDPV in the population of *M. monachus* in Madrid was investigated by PCR in cloacal swab samples of 73 specimens, as well as in the 28 samples that were submitted for metagenomic analysis. None of the captured individuals showed signs of disease, and all PCR results were negative, except for one of the 28 samples that had been included for NGS.

## 4. Discussion

The population of exotic invasive species such as monk parakeets has increased in several European cities and most urban areas along the Mediterranean coast. Madrid is the area with the highest concentration of monk parakeets in Spain [7]. They constitute a severe threat to native bird species, habitats, or ecosystems, and therefore the study of their virus is of particular interest.

In this study, we have detected and characterized in cloacal samples from urban populations of *M. monachus* a novel genome of dependoparvovirus, named Sp_PsDPV. The virus has a classic parvovirus structure, and its genomic organization and phylogenetic inference place it in the *Dependoparvovirus* genus. The genomic organization is similar to that of the closest dependoparvovirus relative, also identified in the feces of a Chinese wild psittacid (Ch_PsDPV) [34], although it is a partial sequence that has not allowed a complete comparison of the genome. In both viruses, three promoters have been identified in positions similar to those described in the genus, although the first promoter of Sp_PsDPV (comparable to P_5_) lacks a canonical TATA-box. The analysis of this region shows that it might belong to a CpG island-associated category of promoters, most of them related to RNA polymerase II [38]. However, no CpG islands were founded in Ch_PsDPV.

The phylogenetic inference based on the Repprotein groups the genera *Chaphamaparvovirus*, *Aveparvovirus*, and *Dependoparvovirus* in three independent clusters in accordance with previous studies [12,15,40,41,42]. According to the rules for the species demarcation criteria proposed by the ICTV, new parvovirus species can be considered when the replication initiator proteins (NS1 or Rep1, 68 or 78) share <85% of amino acid sequence identity [43]. Sp_PsDPV and Ch_PsDPV shared 80% Rep protein sequence identity with each other, but only 64% with their closest relatives, the two recognized species, DPV and AAAV, and other unclassified members. Both viruses were included in a highly supported clade in a phylogenetic analysis of Rep, distant romf unclassified dependoparvoviruses identified in Passeriformes, Anseriformes, and Piciformes. Likewise, the topology is coincident using VP1 protein (Supplementary Figure S2). These genetic distances and the existence of a monophyletic cluster suggest that Sp_PsDPV conforms to a new species of dependoparvovirus, different from the two species recognized by ICTV, DPV, and AAAV1. We propose that the novel species could tentatively be named Psittacid dependoparvovirus 1 (PsPDV-1).

The genus *Dependoparvovirus* includes adeno-associated viruses from many different species. With the notable exception of goose and duck dependoparvoviruses (which are pathogenic), efficient replication of dependoparvoviruses depends on the presence of a “helper” virus, specifically an adenovirus, herpesvirus, or poxvirus [44]. The Sp_PsDPV described here might be replication-deficient, since all monk parakeets were apparently healthy, and an adenovirus was also found by the NGS analysis.

In our study, metagenomic results came from a pool of 28 monk parakeets. However, the results of the prevalence study in cloacal swabs from 73 individuals of *M. monachus* suggest that the presence of these viruses may be very low in these birds. However, in our study, parvoviral reads represented 5.5% of the total viral reads. The abundance of parvovirus-associated reads in NGS studies of fecal avian viral genome is highly variable, ranging from 1.7% of the eukaryotic viruses [45] to nearly 30% of the total virus reads [34,46]. Nevertheless, several parvovirus taxa may be more represented than others, reaching a prevalence of up to 27% in field surveys [15]. In addition, Psittaciformes has been shown to be one of the bird orders harboring the highest proportion of parvoviruses [34], although their abundance might vary depending on seasonal factors and the age of the birds [42]. To improve the detection of viral families in metagenomic assays, key factors such as the date of capture, the extent of sampling, and the growth stage of the birds should be considered in future studies.

Previous studies have provided evidence of natural genetic recombination in parvoviruses [47], and it may play an important role in the evolutionary process of waterfowl dependoparvoviruses (*Anseriform dependoparvovirus* 1) [48,49,50,51], but no indication of recombination was observed in the Sp_PsDPV and Ch_PsDPV, similar to what has been previously reported [34]. The recombination events usually occur when there is a larger circulating population of viruses closely related to each other [52]. Although this might suggest that it is possible that there are no nearby dependoparvoviruses in the monk parakeets sharing the geographic area where the samples were taken, it would be necessary to analyze a larger number of individuals of *M. monachus* to confirm this possibility.

Though the monk parakeets were apparently healthy, they nevertheless could pose a risk for native birds, to which they could transmit their viruses mainly by the fecal route and affect the health status of indigenous birds with unpredictable consequences. Care must be taken to avoid this situation by deepening our knowledge of the viral genome of this exotic species. Whether these new dependoparvoviruses from psittacine can transmit to other avian hosts needs further investigation.

In summary, we have characterized a possible new species of dependoparvovirus in monk parakeets in Madrid. Despite the low prevalence of this dependoparvovirus in populations of *M. monachus*, there is a risk of introducing new viral species of invasive bird species into new ecosystems. Therefore, these results highlight the importance of scanning the viral geome in invasive populations to prevent and monitor possible cross-transmission to naive native species that would allow the emergence of new diseases and increase zoonotic risk.

## Data Availability

Data can be obtained from the corresponding authors or through the Animal Viruses UCM Research Group available at https://www.ucm.es/animalvirucm/ (accessed on 10 October 2022).

## Supplementary Materials

The following supporting information can be downloaded at: https://www.mdpi.com/article/10.3390/v15040850/s1, Figure S1: Color-coded pairwise identity matrix generated from NS1 (A) and VP1 (B) amino acid sequences of the Sp_PsDPV and representative members of the *Parvoviridae* family; Figure S2: Maximum-likelihood phylogenetic inference of 83 VP1 protein sequences of avian parvovirus; Table S1: NLS sequences of dependoparvovirus; Table S2: Predictions of promoters, splicing, and polyadenylation sites.
