# Supplementary material for: A Novel Dependoparvovirus Identified in Cloacal Swabs of Monk Parakeet (Myiopsitta monachus) from Urban Areas of Spain
Source: Viruses. 2023 Mar 26;15(4):850. doi: 10.3390/v15040850 (PMC10145644; doi:10.3390/v15040850)
Supplement: Supplementary file 1 [file viruses-15-00850-s001.zip › viruses-2244569-supplementary.pdf]

Supplementary Material

A novel dependoparvovirus identified in cloacal swabs of Monk Parakeet (*Myiopsitta monachus*) from urban areas of Spain

Christian Sánchez [1], Ana Doménech [2,3] Esperanza Gomez-Lucia [2,3] José Luis Méndez [4], Juan Carlos Ortiz [5] and Laura Benítez [1,3]*

*** Correspondence:** Corresponding Author: lbenitez@ucm.es

1.1 Supplementary Figures

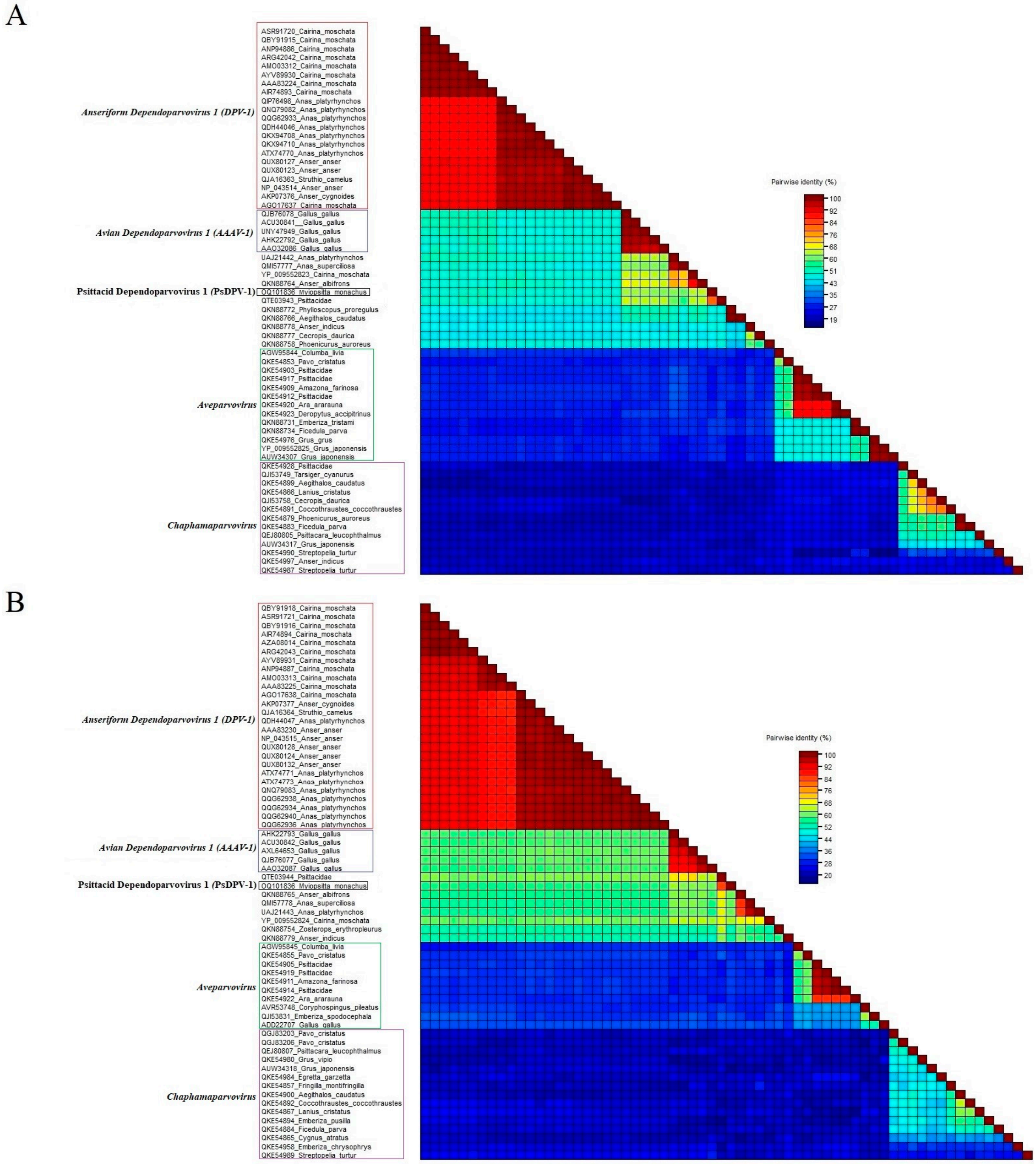

Supplementary Figure S1. Color-coded pairwise identity matrix generated from NS1Rep (A) and VP1 (B) amino acid sequences of the Sp_PsDPV and representative members of the *Parvoviridae* family. The protein pairwise identity was calculated with the SDT v1.2 program and was plotted as color ranges according to the percentages of protein identity. The Sp_PsDPV and Ch_PsDPV strains are enclosed in a box.

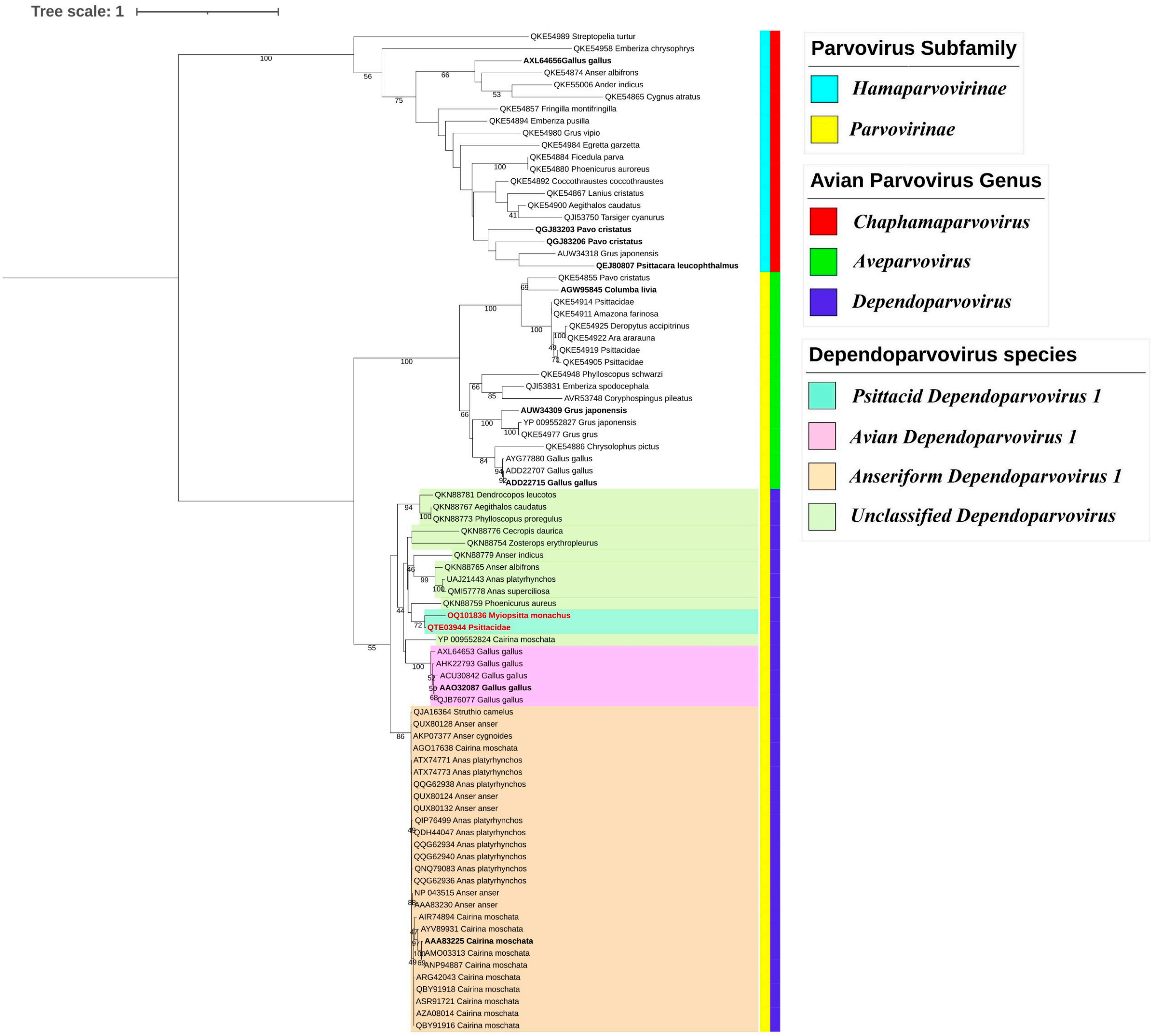

Supplementary Figure S2. Maximum-likelihood phylogenetic inference of 83 VP1 protein sequences of avian parvovirus. The tree was inferred under a Q.pfam+R+F amino acid substitution model with nodal support values based on 1000 bootstrap replicates. The color code for the subfamilies, genus and species used in the vertical bars to the right of the names of the species in the tree is indicated in the legend on the right. Representative sequences of avian parvovirus species are in bold and the dependoparvoviruses from Psittacidae are highlighted in red. Only the support values above 50 are displayed.

1.2 Supplementary Tables

Supplementary Table S1. NLS Sequences of dependoparvovirus

Genus	Species	VP1 Protein GenBank	NLS sequence	Position
Dependoparvovirus	*Anseriform dependoparvovirus 1*	QBY91918	QQSQSVSTDRKPRRKDNNRGFVLPG	33 and 57
Dependoparvovirus	*Anseriform dependoparvovirus 1*	ASR91721	QSQSVSTDRKPQRKDNNRGFVLP	34 and 56
Dependoparvovirus	*Anseriform dependoparvovirus 1*	QBY91916	QSQSVSTDRKPQRKDNNRGFVLP	34 and 56
Dependoparvovirus	*Anseriform dependoparvovirus 1*	AIR74894	QSQSVSTDRKPQRKDNNRGFVLP	34 and 56
Dependoparvovirus	*Anseriform dependoparvovirus 1*	AYV89931	QSQSVSTDRKPQRKDNNRGFVLP	34 and 56
Dependoparvovirus	*Anseriform dependoparvovirus 1*	ANP94887	QSQSVSTDRKPQRKDNNRGFVLP	34 and 56
Dependoparvovirus	*Anseriform dependoparvovirus 1*	AMO03313	QSQSVSTDRKPQRKDNNRGFVLP	34 and 56
Dependoparvovirus	*Anseriform dependoparvovirus 1*	ARG42043	QSQSVSTDRKPQRKDNNRGFVLP	34 and 56
Dependoparvovirus	*Anseriform dependoparvovirus 1*	AZA08014	QSQSVSTDRKPQRKDNNRGFVLP	34 and 56
Dependoparvovirus	*Anseriform dependoparvovirus 1*	QOE76062	Not found	Not found
Dependoparvovirus	*Anseriform dependoparvovirus 1*	AQV09495	Not found	Not found
Dependoparvovirus	*Anseriform dependoparvovirus 1*	QKX94711	Not found	Not found
Dependoparvovirus	*Anseriform dependoparvovirus 1*	QKX94709	Not found	Not found
Dependoparvovirus	*Anseriform dependoparvovirus 1*	ATX74771	Not found	Not found
Dependoparvovirus	*Anseriform dependoparvovirus 1*	QBC88390	Not found	Not found
Dependoparvovirus	*Anseriform dependoparvovirus 1*	QQG62938	Not found	Not found
Dependoparvovirus	*Anseriform dependoparvovirus 1*	QQG62934	Not found	Not found
Dependoparvovirus	*Anseriform dependoparvovirus 1*	QQG62940	Not found	Not found
Dependoparvovirus	*Anseriform dependoparvovirus 1*	QQG62936	Not found	Not found
Dependoparvovirus	*Anseriform dependoparvovirus 1*	QNQ79083	Not found	Not found
Dependoparvovirus	*Anseriform dependoparvovirus 1*	ATX74773	Not found	Not found
Dependoparvovirus	*Anseriform dependoparvovirus 1*	ANX99769	Not found	Not found
Dependoparvovirus	*Anseriform dependoparvovirus 1*	ANN12566	Not found	Not found
Dependoparvovirus	*Anseriform dependoparvovirus 1*	ACE95855	Not found	Not found
Dependoparvovirus	*Anseriform dependoparvovirus 1*	QUX80128	Not found	Not found
Dependoparvovirus	*Anseriform dependoparvovirus 1*	QUX80124	Not found	Not found
Dependoparvovirus	*Anseriform dependoparvovirus 1*	QUX80132	Not found	Not found
Dependoparvovirus	*Anseriform dependoparvovirus 1*	QDH44605	Not found	Not found
Dependoparvovirus	*Anseriform dependoparvovirus 1*	ACE95849	Not found	Not found
Dependoparvovirus	*Anseriform dependoparvovirus 1*	QOW77864	Not found	Not found
Dependoparvovirus	*Anseriform dependoparvovirus 1*	QOW77868	Not found	Not found
Dependoparvovirus	*Anseriform dependoparvovirus 1*	QOW77866	Not found	Not found
Dependoparvovirus	*Anseriform dependoparvovirus 1*	AIX09857	Not found	Not found
Dependoparvovirus	*Anseriform dependoparvovirus 1*	AGG56528	Not found	Not found
Dependoparvovirus	*Anseriform dependoparvovirus 1*	AGO17638	Not found	Not found
Dependoparvovirus	*Anseriform dependoparvovirus 1*	ACE95851	Not found	Not found
Dependoparvovirus	*Anseriform dependoparvovirus 1*	QDH44047	Not found	Not found
Dependoparvovirus	*Anseriform dependoparvovirus 1*	AAA83230	Not found	Not found
Dependoparvovirus	*Anseriform dependoparvovirus 1*	NP_043514	Not found	Not found
Dependoparvovirus	*Anseriform dependoparvovirus 1*	AKP07377	Not found	Not found
Dependoparvovirus	*Anseriform dependoparvovirus 1*	QJA16364	Not found	Not found
Dependoparvovirus	*Anseriform dependoparvovirus 1*	QIP76499	Not found	Not found

Dependoparvovirus	Avian dependoparvovirus 1	UNY47950	VDDFFPKKKKAKTEQGKAPAQA	158 and 179
Dependoparvovirus	Avian dependoparvovirus 1	AHK22793	VDDFFPKKKKAKTEQGKAPAQT	158 and 179
Dependoparvovirus	Avian dependoparvovirus 1	ACU30842	VDDFFPKKKKAKTEQGKTPAQT	158 and 179
Dependoparvovirus	Avian dependoparvovirus 1	AXL64653	VDDFFPKKKKAKTEQGKAPAQT	158 and 179
Dependoparvovirus	Avian dependoparvovirus 1	QJB76077	EPRLPDTPPQTPKKNKKPRKERPSDGAEDP	158 and 187
Dependoparvovirus	*Psittacid Dependoparvovirus 1*	QTE03944	Not found	Not found
Dependoparvovirus	*Psittacid Dependoparvovirus 1*	**OQ101836**	VDDFFPKKKKAKTNHTEPEKSS	155 and 176
Dependoparvovirus	*Unclassified dependoparvovirus*	QKN88779	Not found	Not found
Dependoparvovirus	*Unclassified dependoparvovirus*	QKN88776	Not found	Not found
Dependoparvovirus	*Unclassified dependoparvovirus*	QKN88759	Not found	Not found
Dependoparvovirus	*Unclassified dependoparvovirus*	QKN88754	Not found	Not found
Dependoparvovirus	*Unclassified dependoparvovirus*	QKN88781	Not found	Not found
Dependoparvovirus	*Unclassified dependoparvovirus*	QKN88773	Not found	Not found
Dependoparvovirus	*Unclassified dependoparvovirus*	QKN88767	Not found	Not found
Dependoparvovirus	*Unclassified dependoparvovirus*	QKN88765	Not found	Not found
Dependoparvovirus	*Unclassified dependoparvovirus*	QMI57778	AGAPKPKPNQQHQDRGEPKD	31 and 50
Dependoparvovirus	*Unclassified dependoparvovirus*	YP_009552824	Not found	Not found
Dependoparvovirus	*Unclassified dependoparvovirus*	UAJ21443	EAGAPKPKPHQQHQNRSDAKD	30 and 50

Supplementary Table S2. Predictions of promoters, splicing and polyadenylation sites

Species	GenBank	Prediction	Software	Sequence	Value
Psittacid Dependoparvovirus 1	**OQ101836**	P5	NNPP	-	-
Psittacid Dependoparvovirus 1	**OQ101836**	P19	NNPP	CCCGCGTGC**TTAAA**TGCACGCCTCCGAGCGGAAATTGCGG**A**GACGCACTT	0.9
Psittacid Dependoparvovirus 1	**OQ101836**	P40	NNPP	CCCCGGAAGC**TATA**AAAGAGGCCTTTGGAACCGCGAGTCC**T**CCCATTCGT	0.99
Psittacid Dependoparvovirus 1	MW046511	P5	NNPP	-	-
Psittacid Dependoparvovirus 1	MW046511	P19	NNPP	CGCGATAAATCA**TATA**TTCCGGCCTACTTGATTCCGAAAC**A**GCAACCGGA	0.94
Psittacid Dependoparvovirus 1	MW046511	P40	NNPP	CCCCTGAGAC**TATA**AAAAGAGCAACGACTTCCGCGTTTCA**G**TCAGTCTCG	0.98
Anseriform Dependoparvovirus 1	U22967	P5	NNPP	CTAGGAGAGTG**TATA**AGGAGAGCTTTTTCCGGTTGCATTC**A**TTCGTTGCT	0.91
Anseriform Dependoparvovirus 1	U22967	P19	NNPP	GGGAAACAGGTAAAAATCCCGGATTGGTTTTC**TATA**ACTA**A**AACCAAACG	0.98
Anseriform Dependoparvovirus 1	U22967	P40	NNPP	TAGAGGAGA**TATTA**AGAGCGAGCCCTGAGCTCTTTGCTTC**A**GTTGCTCCT	0.78
Avian Dependoparvovirus 1	AY186198	P5	NNPP	ACGTAAACAAA**TATA**AGACGGCGCCACACGGCGCTGCGTC**A**TACGCGCGC	1
Avian Dependoparvovirus 1	AY186198	P19	NNPP	TGCCCGAG**TATA**TAAAAGCGTGCTTGCACCGAGAACTGCG**T**GCCAGTCTC	0.98
Avian Dependoparvovirus 1	AY186198	P40	NNPP	CGGGGAAGGC**TATA**TAAGCCCGACAAAGCGGCCCGCGCTC**G**CAGAGCAGC	1
Psittacid Dependoparvovirus 1	**OQ101836**	Splicing Donnor	NetGene2	ACCTCGACAG^**GT**ATGAACGC	0.93
Psittacid Dependoparvovirus 1	**OQ101836**	Splicing Acceptor	NetGene2	TCTGCCGC**AG**^ACGCAGTTCC	0.18
Psittacid Dependoparvovirus 1	MW046511	Splicing Donnor	NetGene2	AACTCGACAG^**GT**ACGAACGA	0.93
Psittacid Dependoparvovirus 1	MW046511	Splicing Acceptor	NetGene2	ATCAAAAA**AG**^GAATTGTTAA	0.18
Anseriform Dependoparvovirus 1	U22967	Splicing Donnor	NetGene2	GGAGTATCAG^**GT**ACGCTGTG	0.67
Anseriform Dependoparvovirus 1	U22967	Splicing Acceptor	NetGene2	ACTTTTTT**AG**^AGAAATTTGA	0.16
Avian Dependoparvovirus 1	AY186198	Splicing Donnor	NetGene2	TTCCCACCAG^**GT**ATCGTATC	0.74
Avian Dependoparvovirus 1	AY186198	Splicing Acceptor	NetGene2	GCGATTCC**AG**^ATTGGTTGGA	0.18
Psittacid Dependoparvovirus 1	**OQ101836**	PolyA	PolyApred	AATAAA	0.37
Psittacid Dependoparvovirus 1	MW046511	PolyA	PolyApred	-	-
Anseriform Dependoparvovirus 1	U22967	PolyA	PolyApred	AATAAA	0.7
Avian Dependoparvovirus 1	AY186198	PolyA	PolyApred	AATAAA	0.05
