# Supplementary figures and images for: A Novel Dependoparvovirus Identified in Cloacal Swabs of Monk Parakeet (Myiopsitta monachus) from Urban Areas of Spain
Source: Viruses. 2023 Mar 26;15(4):850. doi: 10.3390/v15040850 (PMC10145644; doi:10.3390/v15040850)

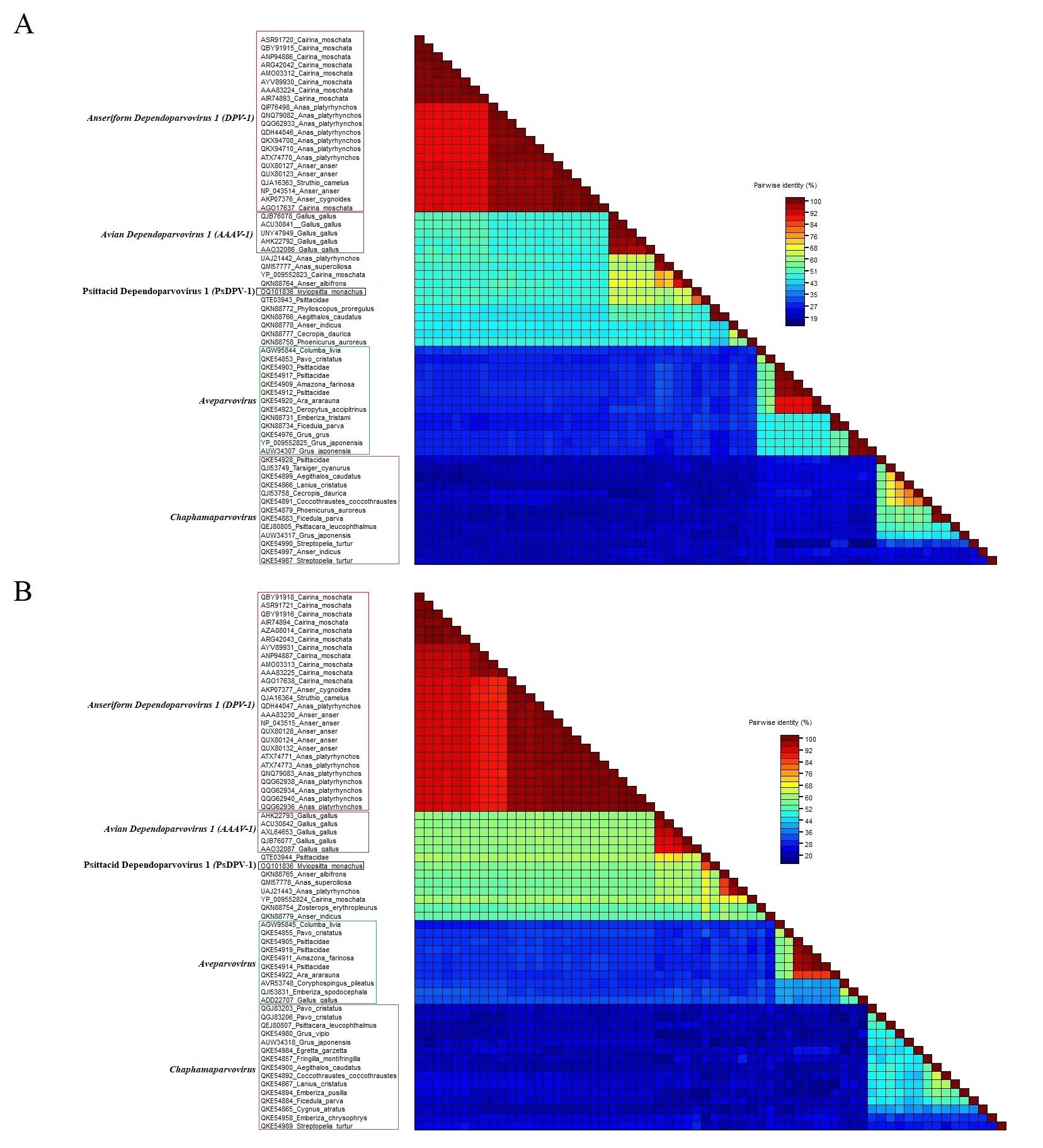

Supplement: Supplementary file 1 [file viruses-15-00850-s001.zip › Supplementary Figure S1.jpg]

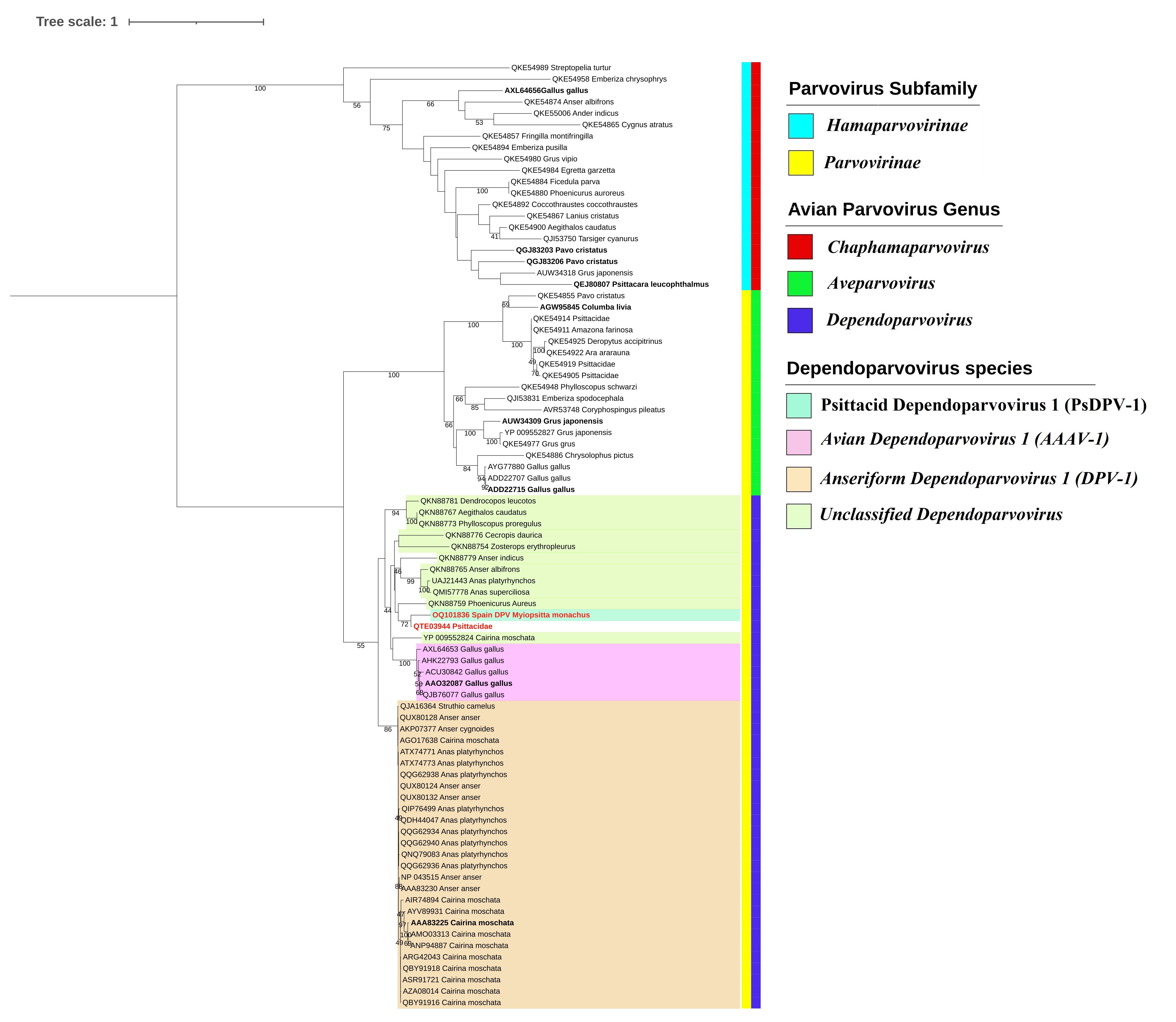

Supplement: Supplementary file 1 [file viruses-15-00850-s001.zip › Supplementary Figure S2.jpg]
